# The Week's Work

**Published:** 1909-12-04

**Authors:** 


### The Week's Work.

### THE HOSPITAL SUNDAY FUND.

We draw attention to the report of the meeting of the Council of the Metropolitan Hospital Sunday Fund which appears on p. 280. Sir William Church's proposal in reference to Mr. Alfred Willett's services voices the feelings of all the members, who cannot but regret that Mr. Willett has retired from the Distribution Committee, on which he served for more than thirty years. His influence on the Committee was unifying and valuable, and his absence will be felt in the future. A far less unanimous feeling of regret was voiced by Mr. Lionel Lewis in his notice of motion for the alteration of the law of the constitution so as to provide for the election of the Distribution Committee by the constituents instead of by the Council as hitherto. As the secretary pointed out, the acceptance of such a motion will radically alter the procedure which has been followed for the last thirty-seven years. Moreover, it will introduce into the deliberations of the Committee factors and influences which are at present outside. There can be no hesitation, therefore, in condemning it. Hitherto the Distribution Committee has carried on its work impartially, honestly, and well, and its awards, as was pointed out at the meeting, have never been seriously questioned. In the circumstances we do not think that the constituents will feel justified, or that they will deem it wise, to take the duties of distribution out of the hands of the Committee appointed on a plan which has proved so satisfactory.

### A FOOD STANDARD.

Dr. Oldfield, at a meeting convened by the National Pure Food Association held on Tuesday the 30th ult., stated that the Local Government Board was opposed to the use of boric acid as a preservative in milk. Canon Horsley, Mayor of Southwark, in supporting the establishment of a food standard by Act of Parliament, explained that the ultimate objects in view were (1) to compel local authorities to administer efficiently the Sale of Foods and Drugs Acts; (2) to improve the existing law relating to adulteration; (3) and to set up standards with which all foods sold to the public should comply. Canon Horsley was severe on the shopkeeper as a borough councillor, and suggested that in many of the districts where the Food and Drugs Acts were inefficiently administered the cause was to be found in a preponderance of shopkeepers on the local authority responsible for the administration of the law. We agree with another speaker, Col. Cassal, that no form of repressive legislation will secure a food supply of high quality. The remedy is to educate the children in schools and the public generally to a point where they can estimate for themselves the quality of all food before they purchase it. Why does not every man before he marries satisfy himself that his future wife possesses this knowledge and take steps to acquire it himself?

### THE NORTH STAFFORDSHIRE INFIRMARY.

The annual meeting of governors on the 25th ult. was largely attended. It is satisfactory to note that the discussion throughout kept well in mind that the best interests of the Infirmary must be the main consideration with all parties. The first business was the appointment of a President, and Col. A. H. Heath was unanimously elected. He stated that in accepting election he welcomed the fact that at last an interest had been awakened in the institution and in everything which belonged to it and affected it, which had never been aroused before. Mr. Wade, the Mayor of Hanley, and other independent governors voiced the demand for investigation and reconstruction. In the result it was decided to await the reports of two sub-committees already appointed, one on the rules and one on buildings and reconstruction. Ultimately certain governors to fill vacancies were elected on the committee, and a consulting committee of ten independent governors was appointed to co-operate with the general committee and to sit with them. When this joint committee had received the reports of the sub-committees and considered them, it was agreed to call a second meeting of the governors, with the understanding that the members of the existing committee would place themselves in the hands of that meeting for re-election or otherwise, as the governors might then decide. We welcome the appointment of Col. Heath as President, for we take it to mean that in due course the honorary medical staff will be adequately increased, the out-patient department reconstructed, and the rules and regulations brought up to date, so that the governors may fully represent all classes of subscribers. Mr. Spanton, F.R.C.S., who has been appointed chairman of the building sub-committee, though in a minority for some months, has strenuously advocated reform, and we shall look forward to the report of his committee with especial interest.

### METHODS AND MEN.

A reporter for a great newspaper, with keen and developed powers of observation, after some years should be a gold mine to his Editor. In the course of his work he picks men up in their ordinary avocations where they are best and least known, and when they spread themselves on public platforms in speech. Nowadays men in public life choose one of two methods. Either they suppress the *ego* altogether and make their individuality felt by the quality and volume of the work which they do in connection with anything they touch, or they get their name associated with the management of certain institutions or movements, and remind their hearers of the fact in nearly every speech they make. In brief, the policies are: Work and self-suppression *versus* self-advertisement and some work of the half-time principle. The experienced reporter, observing and knowing his men, especially if he attends a charity dinner now and then, should be capable of producing soliloquies on men as he finds them which should sell out every edition of his paper. It is amazing how ignorant people who reside almost under the shadow of a great hospital may be of even its strong points. Owing to the success of the lie in politics, lying, we fear, may become general if the minds of the best men are not brought to bear upon this national danger. We were told in good faith by the head of a municipality that one of the best administered London hospitals in his district is grossly extravagant, and that it has received no grant from the King's Fund in consequence. This statement has been uttered publicly many times, and has been driven home to the mind of the innocent Mayor. In fact, this hospital from the outset has received a splendid grant each year from the King's Fund, and is deservedly recognised as one of the most economically and best administered of Metropolitan hospitals.

### A SECRETARY'S GOLD MINE.

The truth is hospital secretaries have got to wake up and assert themselves and the claims of their hospitals. The present season is ripe for a new departure which may prove a gold mine in practice. Let every voluntary hospital issue its own almanac or diary, "The —— Hospital Diary," and let it contain every point of importance affecting the particular hospital and its administration: the King's Fund grant; the cost per bed; the steady fall in such cost, and in that of each out-patient or in-patient; economies effected in various departments; the methods of checking abuse; the needs of the particular hospital; the opportunities it offers for personal service; the claims it has upon local residents; its work; its influence; its successes, failures, sorrows, and so forth. Let this almanac or diary be sent free as a New Year's gift to every resident within the district which each hospital provides for. The first result should be an educated public opinion which would destroy the liars, and the next a wider area of givers and supporters for each voluntary hospital. We have gone into the question of cost, based upon a model diary, and find it is small enough to make the scheme a good business proposition. It therefore merits the careful consideration of the managers of the larger voluntary hospitals throughout the country which are administered with knowledge and sound judgment. The management of a voluntary hospital must be up-to-date, or the hospital revenues must suffer.

### THE VOLUNTARY SYSTEM IN THE COLONIES.

The hospital question in South Africa is one of the burning questions of the day which will have to be tackled sooner or later. Hitherto the State and the public have both put its consideration aside; but once the different colonies have been merged into a homogeneous whole by the realisation of union, the question is likely to be taken in hand and dealt with. Meanwhile there is some speculation as to the manner in which it will be dealt with. Dr. Ronald Mackenzie read an interesting paper on the subject before the recent South African Medical Congress, but touched the fringe of the subject only, and hesitated to declare himself frankly. He put forward a summary of the voluntary and the State systems, with a strong leaning towards the former, but hinted that in the colonies the tendency is towards welcoming the latter. In discussing the State system, he outlined its advantages to the general practitioner, and pointed out that under the voluntary system the better classes show an increasing demand for hospital treatment. Under the State system, apparently, he imagines that this will not be the case, since the well-to-do patient would scruple to enter a ward where the beds were supported out of the rates. For that reason Dr. Mackenzie would prefer that all the patients in a State-supported hospital should be free patients—a rule which is certainly not enforced in Germany or Austria, where payment is exacted in all cases except in those where the patients are obviously destitute.

### STATE AID AND NATIONALISATION.

In the State system Dr. Mackenzie, it is interesting to notice, sees the thin end of the wedge of ultimate nationalisation of the medical profession. While there can be little question that such countries as Germany, where the State system in its purest form has now had an extended trial, are nearer nationalisation, so far as the medical profession is concerned, than either England, where the system exists only for the fever hospitals, or the United States, where a compromise between the two systems has been effected, it is scarcely possible to conceive such nationalisation while medicine remains on its present footing. This is entirely the view of our professional colonial contemporaries, who, in discussing Dr. Mackenzie's paper, point out that "it is doubtful whether the nationalisation of hospitals would not at present be one of those regrettable leaps in the dark sometimes taken by new countries under the impression that they are exhibiting a creditable go-ahead example to older and more thoughtful communities." The *Transvaal Medical Journal*, in a leading article on the subject, remarks as follows: "We are inclined to believe that the ultimate solution of the hospital question may be found in the establishment of State hospitals, with an honorary staff, for non-paying patients. This would necessitate an exact definition of what constitutes eligibility for admission to a free hospital: the tax-payer would not tolerate the admission of any but those who are unable to meet the expense of outside treatment. The income limit by itself is not always a reliable standard, in a new country especially. Whilst all men should earn fair incomes, many may not earn enough to keep a family and at the same time support the pecuniary strain involved in an attack of enteric fever or an operation for renal calculus. . . . . The business of meeting the demand for hospital treatment by those who are able to pay for it should be left to private enterprise." The system thus outlined will hardly commend itself to anyone who has any experience of the working of the various systems, for it is at the best an attempt to compromise between two virtually antagonistic principles.

